# Cerebellar tumefactive demyelination in MOGAD: a case report on diagnostic challenges and immunotherapeutic strategy

**DOI:** 10.3389/fimmu.2025.1729641

**Published:** 2026-01-19

**Authors:** XiaoDan Zheng, JiaYue Zhang, DaWei Li, Bo Yuan

**Affiliations:** Department of Neurology, The Fourth People’s Hospital of Shenzhen, Shenzhen, China

**Keywords:** myelin oligodendrocyte glycoprotein antibody-associated disease, diagnosis, differential diagnosis, MRI, therapy, tumefactive demyelination

## Abstract

Tumefactive demyelination is a rare phenotypic subtype of myelin oligodendrocyte glycoprotein (MOG) antibody-associated disease (MOGAD) and poses significant diagnostic challenges due to substantial clinical and radiological overlap with intracranial neoplasms and other demyelinating conditions. This mimicry frequently leads to misdiagnosis and subsequent inappropriate therapeutic interventions and delay in treatment, thereby increasing the risk of adverse clinical outcomes. We present the case of a 31-year-old male with a prior history of MOG-associated optic neuritis (ON) who developed acute-onset dizziness and gait instability evolving over five days. Brain magnetic resonance imaging (MRI) demonstrated an atypical mass-like lesion in the left cerebellar hemisphere with extension to the middle cerebellar peduncle. Serum testing showed MOG-IgG positivity at 1:100 titer (live cell-based assay) with negative AQP4-IgG. Following timely pulse corticosteroid therapy, the patient showed marked symptomatic improvement and received sequential maintenance of oral corticosteroid for six months. A follow-up MRI revealed complete resolution of abnormalities, and no recurrence was observed during the 21-month follow-up period. This case highlights the critical importance of including rare tumefactive MOGAD in the differential diagnosis of mass-like lesions, thereby avoiding the potential morbidity associated with misdiagnosis and delayed treatment. It also underscores the role of maintenance therapy in reducing relapses and preventing disability accumulation.

## Introduction

Myelin oligodendrocyte glycoprotein antibody-associated disease (MOGAD) is an inflammatory demyelinating condition of the central nervous system (CNS) with a diverse clinical spectrum, including optic neuritis (ON), transverse myelitis (TM), acute disseminated encephalomyelitis (ADEM), brainstem encephalitis, aseptic meningitis, cortical encephalitis, and tumefactive demyelinating lesions (1–4). Among these, the tumefactive demyelinating lesions represent a rare phenotypic subtype that poses a significant diagnostic challenge by mimicking a mass-like lesion on imaging. This resemblance frequently leads to misdiagnosis as a CNS neoplasm, especially glioma, potentially prompting unnecessary invasive procedures such as brain biopsy and thereby exacerbating the disease burden and delaying appropriate therapeutic intervention. Although radiologic distinctions between umefactive demyelinating diseases like multiple sclerosis (MS) and intracranial gliomas have been explored, the imaging profile of tumefactive MOGAD is distinct from that of MS or other demyelinating entities, and its differentiation from intracranial gliomas remains rarely reported.

The identification of characteristic imaging features in tumefactive lesions is a critical diagnostic clue in MOGAD, warranting MOG-IgG testing to facilitate a timely and accurate diagnosis. Prompt therapeutic intervention is essential, as MOGAD is frequently relapsing, with recurrence risk correlating with antibody titers and persistent seropositivity. Early treatment initiation during the initial attack can promote seroconversion to negative status, thereby reducing the risk of relapse and clinical deterioration. Moreover, maintenance immunotherapy is fundamental to modifying the long-term disease course and preventing the accumulation of permanent neurological disability. Nonetheless, significant challenges in diagnosis and management remain in clinical practice (5).

We hereby present a representative case that highlights the crucial role of characteristic imaging features in diagnosing tumefactive MOGAD and underscores the importance of early and maintenance immunotherapy for improving long-term clinical outcomes.

## Case presentation

**Figure 1:**
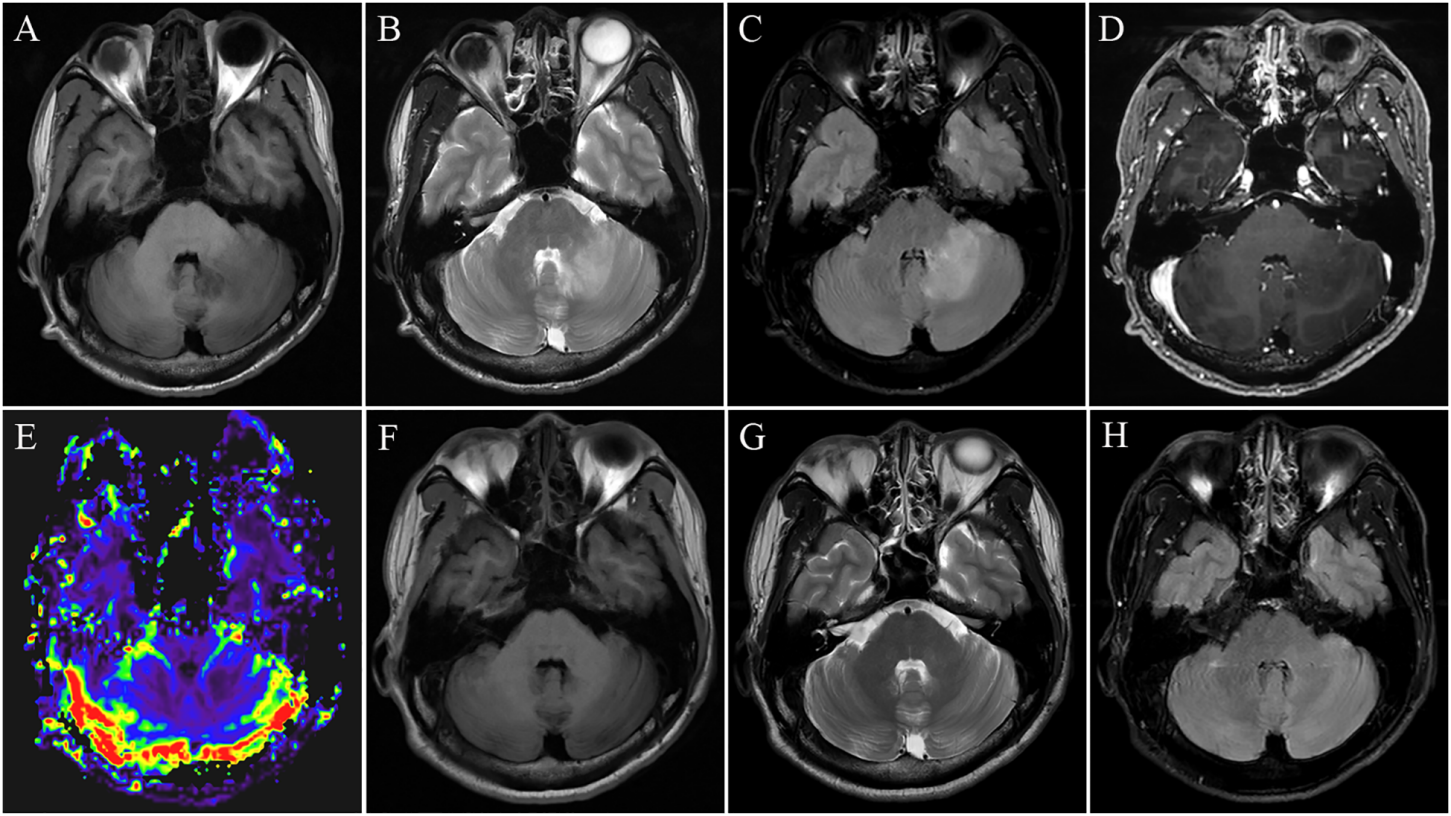
Initial brain magnetic resonance imaging (MRI) demonstrated a mass-like lesion in the left cerebellar hemisphere extending to the middle cerebellar peduncle. The lesion was hypointense on T1-weighted images **(A)**, hyperintense on T2-weighted **(B)** and fluid-attenuated inversion recovery (FLAIR) sequences **(C)**, with scattered patchy and linear enhancement on post-contrast T1-weighted imaging **(D)** and reduced relative cerebral blood volume (rCBV) on perfusion-weighted imaging (PWI) **(E)**. A follow-up MRI performed five months later showed complete resolution of these abnormalities on all sequences without any residual signal changes **(F–H)**.

A 31-year-old male presented with a five-day history of progressively worsening dizziness and gait instability. He had been diagnosed with MOG antibody-associated ON nine months prior, following an episode of monocular visual loss (serum MOG antibody titer 1:100), which resolved after corticosteroid therapy. Due to poor adherence, he did not initiate any long-term maintenance immunotherapy. A follow-up serum titer one month before this presentation was 1:32. Neurological examination revealed horizontal gaze-evoked nystagmus with left gaze predominance, as well as left-sided limb dysmetria accompanied by intention tremor. Brain magnetic resonance imaging (MRI) revealed a large, mass-like, “fluffy” lesion in the right cerebellar hemisphere extending into the middle cerebellar peduncle (**Figure 1**). The lesion was hypointense on T1-weighted imaging (T1WI) (**Figure 1A**) and hyperintense on both T2-weighted (**Figure 1B**) and fluid-attenuated inversion recovery (FLAIR) sequences (**Figure 1C**). No restricted diffusion was observed on diffusion-weighted imaging (DWI), and there was a notable absence of mass effect or perilesional edema. Contrast-enhanced T1WI showed minimal, scattered patchy and linear enhancement (**Figure 1D**), while perfusion-weighted imaging (PWI) demonstrated reduced relative cerebral blood volume (rCBV) within the lesion (**Figure 1E**). Cerebrospinal fluid (CSF) analysis demonstrated opening pressure 130 mmH_2_O (normal range: 80~180 mmH2O), leukocytosis 9 cells/μL (normal range: 0~8/μL) with 100% mononuclear, normal protein levels, and negative oligoclonal bands. Serum testing confirmed MOG-IgG positivity at 1:100 titer (live cell-based assay) with negative AQP4-IgG. A diagnosis of MOGAD manifesting as a tumefactive demyelinating lesion was made. Treatment with high-dose intravenous methylprednisolone led to significant clinical improvement within 72 hours and complete resolution of nystagmus and ataxia prior to discharge.

The patient was maintained on oral prednisone (1 mg/kg/day), which was tapered by 5mg every two weeks over a total course of 6 months. Repeat brain MRI at 5 months demonstrated complete radiological resolution of these abnormalities on all sequences without any residual signal changes (**Figures 1F–H**). Serological follow-up showed a decline in the MOG-IgG titer from 1:100 to 1:10. No clinical relapses occurred over a 21-month follow-up period.

## Discussion

Tumefactive demyelination is characterized by large (>2 cm) mass-like demyelinating lesions in the brain and can present with a range of symptoms including impaired consciousness, cognitive deficits, seizures, and focal neurological signs (6). While frequently occurring MS, this entity is also associated with other demyelinating disorders, such as ADEM, AQP4-IgG-seropositive neuromyelitis optica spectrum disorder (NMOSD), and MOGAD (4, 7). Tumefactive demyelination poses a diagnostic challenge due to its radiographic similarity to CNS neoplasms, particularly glioma, and often requires differentiation from cerebral abscess, ischemia, and other inflammatory conditions (6, 8). This diagnostic difficulty is particularly compounded in MOGAD, where the current literature remains largely confined to sporadic case reports, highlighting a significant knowledge gap.

The identification of imaging hallmarks specific to tumefactive MOGAD is fundamental to improving diagnostic accuracy, thereby avoiding misdiagnosis, preventing unnecessary surgical interventions, and enabling timely treatment. Although open-ring enhancement and peripheral diffusion restriction in ring-enhancing lesions are recognized as highly specific features favoring demyelinating diseases over CNS neoplasms (9, 10), these characteristic findings are relatively infrequent in tumefactive MOGAD compared to those in MS (4). The imaging features in our case demonstrate a relatively well-demarcated, “fluffy” lesion in the left cerebellar hemisphere extending into the middle cerebellar peduncle, which notably lacks mass effect, perilesional edema. This radiographic profile is consistent with recently described features of MOGAD-associated tumefactive demyelination (4, 11), thereby offering supportive diagnostic evidence and facilitating differentiation from gliomas. The characteristic involvement of the infratentorial region and middle cerebellar peduncle distinguishes it from classic supratentorial tumefactive demyelination and offers considerable differential diagnostic value (4). The enhancement pattern in our case was characterized by minimal, scattered patchy or linear foci. This reflects mild, incomplete blood-brain barrier (BBB) disruption and contrasts with the intense, nodular or ring-like enhancement typical of high-grade gliomas, which is driven by robust neovascularization. Importantly, hemodynamic features derived from perfusion-weighted imaging (PWI) provide further diagnostic insights. Among these, relative cerebral blood volume (rCBV) is the most frequently utilized parameter (12). In contrast to the characteristic hyperperfusion of CNS neoplasms, the lower rCBV observed in our patient’s lesion supports a pathophysiology distinct from neoangiogenesis (13), and underscores hypoperfusion as a key discriminating feature of tumefactive demyelination. Peripheral hemosiderin rims indicative of iron-laden macrophages, can occasionally be found in MOGAD and serve as a supportive diagnostic feature for demyelination (4).

In addition to conventional MRI sequences, magnetic resonance spectroscopy (MRS) aids in the differentiation of tumefactive demyelination and CNS neoplasms by detecting their distinct metabolic profiles (14). A characteristic metabolic finding in gliomas, particularly in malignant ones, is a marked increase in choline (Cho), indicating enhanced membrane phospholipid turnover resulting from hypercellularity and active proliferation (15). This differs significantly from the Cho levels typically observed in tumefactive demyelination. Another key metabolic distinction is N-acetylaspartate (NAA), which is commonly recognized as an indicator of neuronal integrity. Its reduction is frequently observed in both malignant and benign brain tumors, often due to axonal damage or loss (16). In tumefactive MOGAD, the general absence of diffusion restriction suggests a minimal reduction in N-acetylaspartate (NAA), since previous work indicates that diffusion restriction corresponds to more severe tissue damage, potentially attributable to axonal injury or dense cellular infiltrates in demyelinating diseases (10). The combined pattern of elevated Cho and depressed NAA may serve as a reliable metabolic signature for distinguishing CNS neoplasm from tumefactive demyelination (17). This is supported by the finding that a Cho/NAA ratio cutoff of 1.72 demonstrated promising diagnostic accuracy, although this preliminary finding was less reliable for distinguishing low-grade gliomas (14). Future studies are needed to validate these MRS characteristics, particularly the Cho/NAA ratio, in patients with tumefactive MOGAD. This finding has potential utility in refining differential diagnosis but requires further validation in larger cohorts. Collectively, these radiological features may be useful in informing the differential diagnosis between tumefactive MOGAD and intracranial glioma (**Table 1**). When mass-like lesions demonstrate such features, particularly in young patients with a history of MOGAD, serological testing for anti-MOG IgG should be prioritized thereby facilitating a timely diagnosis while mitigating the risk of unnecessary biopsy.

**Table 1:** MRI in distinguishing tumefactive MOGAD and intracranial glioma.

MRI Features	Tumefactive demyelination	Intracranial glioma
Lesion location and appearance		
Main location	Infratentorial	Supratentorial
	Cerebellum	Cerebral white matter
	Middle cerebellar peduncles	Deep brain: Basal ganglia、Thalamus
	Deep cerebral gray matter	Periventricular region
Other Sites	Brainstem	Cerebellum
	Periventricular region	Brainstem
	Cerebral white matter	Cerebral gray matter
Appearance	fluffy	Heterogeneous patchy
Signal characteristics		
T1WI	Isointense or hypointense	Frequent hypointense
T2WI	Hyperintense	Frequent hyperintense
FLAIR	Hyperintense	Frequent hyperintense
DWI	Absence of restricted diffusion or rare arc peripheral restriction	Absence of restricted diffusion or rare restricted diffusion
T1WI Enhancement	Scattered patchy	Heterogeneous, Nodular, Rim-type
Local effects of the lesion		
Mass effect	Mild	Frequent significant
Perilesional edema	Subtle	Frequent prominent

MRI, magnetic resonance imaging; MOGAD, myelin oligodendrocyte glycoprotein antibody-associated disease; CNS, central nervous system; MRI: magnetic resonance imaging; T1WI, T1-weighted imaging; T2WI, T2-weighted imaging; FLAIR, fluid-attenuated inversion recovery; DWI, diffusion-weighted imaging.

MOGAD typically demonstrates marked corticosteroid responsiveness (18). High-dose intravenous methylprednisolone (1 g daily for 3–5 days) is strongly recommended as first-line acute therapy, which can induce rapid and substantial, often complete, symptomatic recovery in most patients (19). For patients refractory to intravenous methylprednisolone or those with severe presentations, therapeutic options include plasma exchange (five exchanges every other day), immunoadsorption, intravenous immunoglobulins (IVIG; total dose 2 g/kg over 2–5 days), or a combination of plasma exchange followed by intravenous immunoglobulins (19).

It is recognized that MOGAD often follows a relapsing course, leading to significant disability accumulation in a substantial proportion of patients (20). In a retrospective cohort of 240 patients with a median (IQR) disease duration of 3.07 (1.95-6.15) years, 110 patients (45.8%) experienced relapse after a median (IQR) of 0.45 (0.18-1.68) years. Multivariate analysis revealed that both timely initiation of treatment after disease onset and maintenance immunotherapy were independently associated with a reduced risk of relapse. Compared to early treatment (within 4 days of onset), initiating treatment at an intermediate (5–14 days) or late (>14 days) stage was associated with a 2.02-fold (95% CI, 1.10-3.74) and a 2.64-fold (95% CI, 1.43-4.84) increase in the risk of relapse, respectively (5). These findings, in line with previous reports, underscore the critical importance of timely therapeutic intervention to prevent relapses and the accumulation of associated disability in MOGAD (5, 21, 22). Notably, early treatment of the initial attack is critical for promoting seroconversion of MOG-IgG antibody to negativity (5), which strongly predicts a subsequent monophasic disease course, in contrast to the relapsing course associated with persistent seropositivity (23, 24). The present case demonstrates a recurrent demyelinating pseudotumor in a seropositive MOGAD patient with prior ON, thereby further supporting the association between persistent seropositivity and relapsing disease. The mechanism for seroconversion induced by early intervention after the first attack may involve suppressing the proliferation and differentiation of B cells, thereby preventing the formation of autoimmune long-lived plasma and memory B cells, and restricting the antigen-presenting activity of monocytes and dendritic cells through reduced production of activating cytokines (5).

Despite the absence of approved maintenance immunotherapies for reducing relapses in MOGAD, oral corticosteroids, B-cell depleting agents, and azathioprine are the most commonly used in clinical practice (20). For adult patients with MOGAD, a conventional corticosteroid regimen consists of oral prednisone at 1 mg/kg/day for three months, followed by a gradual taper over the subsequent three or more months, depending on the individual’s predisposition to relapse (18, 19). A recent long-term study of 109 patients with MOGAD (median follow-up 6.2 years) demonstrated that initiating prednisone at disease onset and maintaining a dose of ≥12.5 mg/day for at least three months was associated with an 88% reduction in the risk of first relapse (HR 0.12, 95% CI 0.03–0.44). This regimen was well tolerated, with no Grade >3 adverse events reported (25). Although oral corticosteroids have been demonstrated to effectively reduce the risk of disease relapse (25, 26), their long-term use is associated with well-documented toxicities that require careful management, particularly in intolerant patients. In contrast, intravenous immunoglobulin (IVIG) is not an immunosuppressive agent and therefore does not increase the risk of infection or malignancy (27). Several studies have demonstrated that maintenance immunotherapy with IVIG is effective in preventing relapses in patients with MOGAD (28, 29). Furthermore, recent retrospective analyses of regimens initiated at higher doses (0.4-2.0 g/kg) and frequencies (weekly to every 4 weeks) followed by a gradual taper have observed a higher rate of disease relapse in patients who received lower or less frequent IVIG dosing, suggesting a dose-response relationship with fewer relapses at higher doses (29). Evidence from case series and studies in other neuroinflammatory disorders indicates that subcutaneous immunoglobulin (SCIG) has comparable efficacy to IVIG in reducing relapse rates (29, 30). Thus, SCIG represents a viable alternative that potentially offers more stable drug levels by avoiding the peak-trough fluctuations associated with IVIG (31). Rituximab-mediated B-cell depletion reduces relapse risk in MOGAD. However, a significant proportion of patients experience breakthrough relapses. For these individuals, switching to maintenance IVIG or SCIG therapy is associated with a significantly lower risk of subsequent relapse and represents a recommended alternative strategy (32). Beyond immunoglobulin therapy, other immunomodulatory approaches are under investigation or may be considered. Tocilizumab, an anti-IL-6 receptor monoclonal antibody that modulates B-cell maturation and antibody production, has emerged as a promising candidate and is currently being evaluated as a maintenance therapy for refractory or relapsing MOGAD (33, 34). Furthermore, conventional immunosuppressants such as mycophenolate mofetil or azathioprine may serve as alternatives in certain clinical contexts, although robust prospective data specifically supporting their efficacy in MOGAD remain limited. Ultimately, the selection of an alternative agent should be individualized, considering treatment access, patient comorbidities, and tolerability, highlighting the need for further comparative effectiveness studies.

## Conclusion

This case underscores the necessity of including tumefactive demyelination in the differential diagnosis of mass-like intracranial lesions, particularly in patients with a known history of MOGAD. Given its substantial clinical and radiological mimicry of neoplasms, recognizing characteristic imaging findings and promptly MOG-IgG testing are essential to prevent misdiagnosis and unnecessary interventions. In this patient, early pulse corticosteroid therapy followed by maintenance immunotherapy resulted in complete symptomatic and radiological resolution, with no relapse or disability progression over 21 months. Despite this favorable outcome, the absence of unified guidelines for this MOGAD phenotype highlights the urgent need for multicenter studies to establish definitive diagnostic criteria and standardized management protocols for this rare yet clinically significant condition.

## Data Availability

The original contributions presented in the study are included in the article/supplementary material. Further inquiries can be directed to the corresponding authors.
